# Platelet Glycoprotein Ibα Cytoplasmic Tail Exacerbates Thrombosis During Bacterial Sepsis

**DOI:** 10.3390/ijms252111548

**Published:** 2024-10-27

**Authors:** Yue Xia, Chenglin Sun, Kangxi Zhou, Jie Shen, Jiaojiao Li, Qiuxia Huang, Jiahao Du, Sai Zhang, Kang Sun, Renping Hu, Rong Yan, Kesheng Dai

**Affiliations:** Jiangsu Institute of Hematology, Cyrus Tang Medical Institute, Suzhou Medical College of Soochow University, NHC Key Laboratory of Thrombosis and Hemostasis, National Clinical Research Center for Hematological Diseases, Suzhou 215123, China

**Keywords:** platelet activation, GPIbα, inflammation, bacterial infection, PKC

## Abstract

Septic patients, coupling severe disseminated intravascular coagulation (DIC) and thrombocytopenia, have poor prognoses and higher mortality. The platelet glycoprotein Ibα (GPIbα) is involved in thrombosis, hemostasis, and inflammation response. However, it remains unclear whether the GPIbα cytoplasmic tail regulates sepsis-mediated platelet activation and inflammation, especially in Staphylococcus aureus (*S. aureus*) and Escherichia coli (*E. coli*) infections. Using a mouse model of *S. aureus*-induced bacteremia, we found that both 10 amino acids of GPIbα C-terminal sequence deficiency and pharmacologic inhibition of protein kinase C (PKC) alleviated pathogenesis by diminishing platelet activation and aggregate formation. Furthermore, the GPIbα cytoplasmic tail promoted the phagocytosis of platelets by Kupffer cells in vivo. The genetically deficient GPIbα cytoplasmic tail also downregulated inflammatory cytokines and reduced liver damage, ultimately improving the survival rate of the septic mice. Our results illustrate that the platelet GPIbα cytoplasmic domain exacerbates excessive platelet activation and inflammation associated with sepsis through a PKC-dependent pathway. Thus, our findings provide insights for the development of effective therapeutic strategies using PKC inhibitor treatment against bacterial infection.

## 1. Introduction

Sepsis is defined as a life-threatening organ dysfunction caused by dysregulated host inflammatory and procoagulant responses to bacterial infection [1]. Among various bacterial pathogens, Staphylococcus aureus (*S. aureus*) is one of the deadliest bacteria worldwide. However, no global public health investments have been directed toward its control [2]. While numerous studies and clinical trials of novel interventions have been conducted, the mainstay treatment for sepsis has been limited to antibiotic administration [3].

Platelets are detached from the cytoplasm of mature megakaryocytes (MKs) in bone marrow [4]. In addition to their vital roles in hemostasis and thrombosis, platelets also regulate the innate immune system against infection and contribute to inflammatory response [5]. Platelets express many receptors including Toll-like receptors, thrombin receptors, complement receptors, and various adhesion molecules [6]. Many bacteria, such as *S. aureus*, can interact with platelet receptors, and their secreted bacterial toxins are capable of triggering intravascular platelet aggregation [7,8]. Excessive platelet activation leads to disseminated intravascular coagulation (DIC) and drives multiple organ dysfunction syndrome (MODS), which is the cause of the high mortality among patients with sepsis [9].

GPIbα is the major ligand-binding subunit of the glycoprotein (GP) Ib-IX, which is involved in regulating platelet function [10,11]. The extracellular domain of GPIbα interacts with the von Willebrand factor (VWF) and thrombin [12]. The cytoplasmic domain of GPIbα contains binding sites for 14-3-3ζ, which is important for platelet function, and filamin A, which attaches the GPIb-IX complex to the membrane skeleton [13,14]. Published evidence suggests that platelet GPIbα or the GPIbα-leukocyte axis participates in inflammation and microvascular coagulation during bacterial infection [15,16,17,18,19]. Yin H et al. found that interleukin-4 receptor (IL-4R)/Ibα mice, lacking the extracellular domain of GPIbα, showed reduced mortality when challenged with lipopolysaccharide (LPS) and that inhibition of GPIb-IX function prevents LPS-induced platelet adhesion to endothelial cells and microvascular thrombosis [20]. Conversely, another report revealed that the absence of the GPIbα extracellular domain (IL-4R/Ibα) did not improve survival in mice of cecal ligation and puncture (CLP)-induced septic shock, suppressed platelet-monocyte/neutrophil interactions, and enhanced cytokine levels when challenged by CLP [21,22]. These studies indicate that the GPIbα extracellular domain could play multiple roles in regulating inflammation and septic thrombosis. However, the role and underlying molecular mechanism of the GPIbα cytoplasmic domain in sepsis-induced systemic inflammation and thrombosis remains unexplored.

**Scheme 1:**
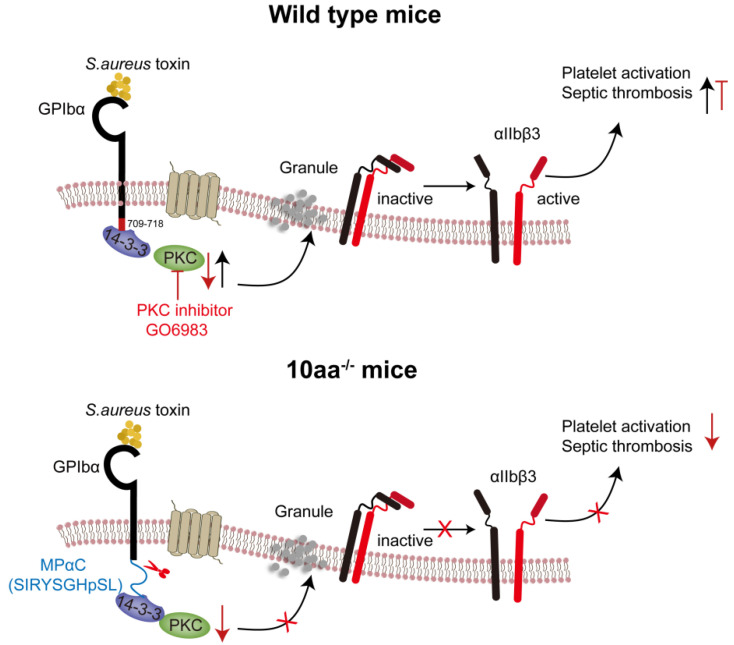
Schematic diagram depicting GPIbα-14-3-3ζ regulation of platelet activation during bacterial infection. Dissociation of 14-3-3ζ from GPIbα results in decreased PKC activity and PKC-mediated integrin α_IIb_β_3_ activation, and granule secretion. MPαC, a peptide corresponding to the C-terminal 14-3-3ζ binding sequence, compensates for the absence of cytoplasmic domain and restores platelet signaling and function.

In the present study, we visualized the intravascular responses to *S. aureus* infection and demonstrated the importance of the GPIbα cytoplasmic tail in platelet clearance, microvascular thrombosis, and the propensity to survive sepsis. Specifically, the GPIbα cytoplasmic tail exacerbated sepsis-induced thrombosis by promoting platelet activation and its inflammatory response through the enhancement of the PKC signaling pathway (Scheme 1). These findings emphasize that PKC inhibition is a potential target for developing new drugs and the therapeutic treatment of thrombocytopenia and thrombosis caused by sepsis.

## 2. Results

### 2.1. Deletion of the GPIbα Cytoplasmic Tail Diminishes Bacteria-Induced Liver Damage, Inflammatory Cytokines, and Mortality

**Figure 1:**
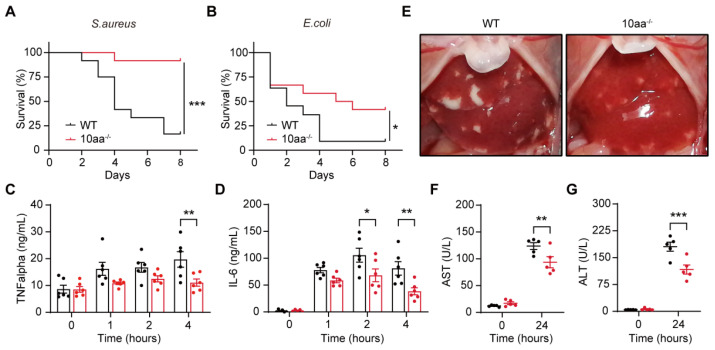
Deletion of the GPIbα cytoplasmic tail diminishes bacteria-induced liver damage, inflammatory cytokines, and mortality. (**A**,**B**) WT and 10aa^−/−^ mice were injected with *S. aureus* (**A**) or *E. coli* (**B**) (1.5 × 10^8^ CFU). The survival rate was observed for up to 8 days (*n*= 12 mice). (**C**,**D**) Blood was collected from WT and 10aa^−/−^ mice before or at 1, 2, and 4 h after *S. aureus* injection i.v. The level of TNFalpha (**C**) and IL-6 (**D**) in plasma was measured by an enzyme-linked immunosorbent assay (*n* = 6 independent experiments). (**E**) 24 h after *S. aureus* injection, the mice were killed and their livers were collected. (**F**,**G**) 24 h after *S. aureus* treatment, plasma from mice was collected and aspartate aminotransferase (AST) (**F**), alanine aminotransferase (ALT) (**G**) concentrations were measured (*n* = 5 independent experiments). Log-rank (Mantel–Cox) test in (**A**,**B**). Two-way ANOVA test in (**C**,**D**,**F**,**G**). Data are shown as mean ± SEM (**C**–**G**). * *p* < 0.05, ** *p* < 0.01, and *** *p* < 0.001.

Increasing studies have shown that platelet GPIbα has important effects on septic inflammation [19,22]. The GPIbα C-terminal domain plays a critical role in GPIbα ligand- or non GPIbα ligand-initiated signal transition and platelet function [23,24]. To investigate the effects of the GPIbα cytoplasmic tail in bacterial sepsis, we used 10aa^−/−^ mice lacking the C-terminal 10 residues of the 14-3-3ζ binding domain of GPIbα. The 10aa^−/−^ mice exhibited phenotypes similar to those of wild-type (WT) mice in the number of hemocytes in the peripheral blood, except for a slightly decreased platelet count (Figure S1A–E). While WT mice succumbed to sepsis induced by intravenous (IV) injection of 1.5 × 10^8^ colony forming units (CFU) of *S. aureus* or *E. coli*, 10aa^−/−^ mice were significantly protected against lethal infection (Figure 1A,B). Sepsis is primarily characterized as systemic inflammation, which exacerbates the development of DIC and septic shock [24,25]. Meanwhile, TNFα or IL-6 levels decreased in 10aa^−/−^ mice post-infection (Figure 1C,D). We then examined organ injury in WT and 10aa^−/−^ mice. Gross pathologic analysis of WT mice livers exhibited large areas of necrosis. The 10aa^−/−^ mice had a markedly lesser degree of injury following infection (Figure 1E), in quantification of plasma aspartate aminotransferase (AST) (Figure 1F) and alanine aminotransferase (ALT) (Figure 1G). Compared with 10aa^−/−^ mice, WT mice exhibited slight splenomegaly (Figure S1F), despite similarities in renal abscesses (Figure S1G) and weight loss (Figure S1H) in both groups. These data indicate that the GPIbα cytoplasmic tail aggravates local inflammation and organ damage.

### 2.2. The GPIbα Cytoplasmic Tail Aggravates S. aureus-Induced Platelet Activation

**Figure 2:**
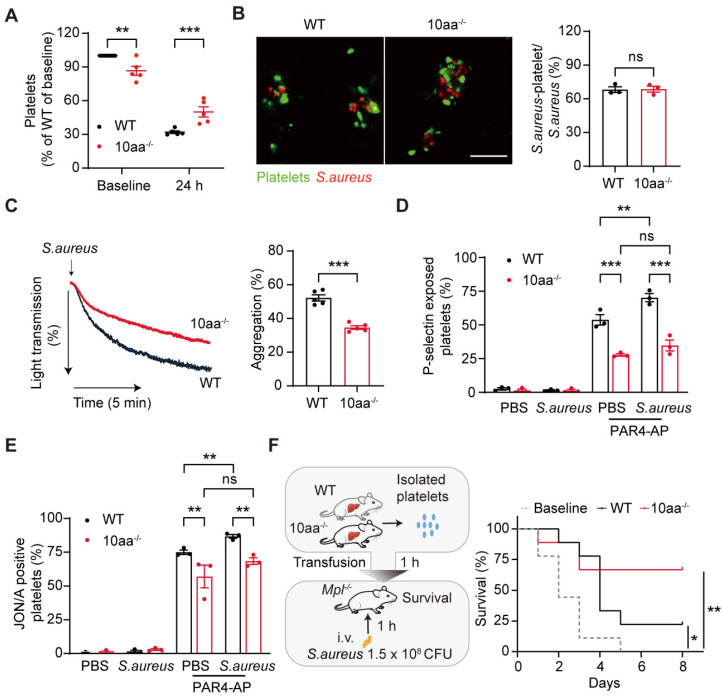
The GPIbα cytoplasmic tail aggravates *S. aureus*-induced platelet activation. (**A**) Platelet counts in the peripheral blood of WT and 10aa^−/−^ mice after *S. aureus* injection i.v. at 24 h (*n* = 5 mice). (**B**) Representative confocal images and quantification of WT and 10aa^−/−^ platelet and *S. aureus* interaction in vivo (*n* = 10 visual fields from 3 mice of each genotype). Scale bar: 10 μm. (**C**) Aggregation of platelets from WT and 10aa^−/−^ treated with fibrinogen in response to *S. aureus* (*n* = 6 independent experiments). (**D**,**E**) Flow cytometric analysis of P-selectin expression (**D**) and JON/A binding (**E**) of WT and 10aa^−/−^ platelets treated with PAR4-AP (*n* = 3 mice). (**F**) The platelets of WT and 10aa^−/−^ mice were transfused into *Mpl*^−/−^ mice or without platelet transfusion (platelets resuspended buffer: MTB as Baseline) following injection with *S. aureus* (1.5 × 10^8^ CFU). Survival rate was monitored for up to 8 days (*n* = 8 mice per genotype). Differences between the two groups were assessed using unpaired two-way ANOVA tests (**A**,**D**,**E**) and unpaired two-tailed Student’s *t*-tests in (**B**,**C**). Log-rank (Mantel–Cox) test in (**F**). Data are shown as the mean ± SEM. * *p* < 0.05; ** *p* < 0.01; and *** *p* < 0.001; ns, no significance.

Coagulation activation and excessive platelet aggregation can lead to sepsis-associated thrombocytopenia and DIC [26]. Thus, a lower platelet count is a sepsis indicator and is associated with a terrible prognosis [27]. In this study, *S. aureus*-induced sepsis in WT mice resulted in a severe platelet count decline at 24 h. In contrast, 10aa^−/−^ mice significantly attenuated the decrease (Figure 2A). The mechanism by which the GPIbα cytoplasmic tail regulates sepsis-induced thrombocytopenia was next investigated. Firstly, the effect of the GPIbα cytoplasmic tail on platelet-bacteria interaction was explored. By incubating WT and 10aa^−/−^ platelet-rich plasma (PRP) with *S. aureus*, or a blooding smear from the WT and 10aa^−/−^ mice infected with *S. aureus*, we found that the deficiency of the GPIbα cytoplasmic tail did not affect platelet-bacteria binding both in vitro and in vivo (Figure 2B and Figure S2A). Thus, the GPIbα cytoplasmic tail does not regulate *S. aureus*-induced sepsis through affecting platelet-bacteria interaction.

Next, we investigated the effect of the GPIbα cytoplasmic tail deletion on *S. aureus*-induced platelet activation. *S. aureus*-induced platelet aggregation was significantly decreased in 10aa^−/−^ platelets in vitro (Figure 2C). To further elucidate the role of the GPIbα cytoplasmic tail in bacteria-induced platelet activation, we assessed P-selectin exposure and GPIIb/IIIa activation on platelets isolated from the mice intravenously injected with 1.5 × 10^8^ CFU of *S. aureus*. While no significant increase in P-selectin levels or GPIIb/IIIa activation (detected by JON/A binding) was observed in circulating platelets after 4 h, platelets from *S. aureus*-injected WT mice were more significantly activated by PAR4-AP stimulation than those from PBS-injected mice (Figure 2D,E), displaying a trend toward increased platelet reactivity in sepsis. Compared with the WT platelets from PBS-injected mice, 10aa^−/−^ platelets from PBS-injected mice showed decreased P-selectin exposure and JON/A binding in PAR4-AP treatment, which is consistent with our previous study [28], and *S. aureus* injection did not further increase 10aa^−/−^ platelet activation (Figure 2D,E). Thus, deletion of the GPIbα cytoplasmic tail inhibits *S. aureus*-induced platelet hyperactivation.

To further prove that the GPIbα cytoplasmic tail in platelets regulates the lifespan of mice with sepsis, we transfused WT and 10aa^−/−^ platelets to *Mpl*^−/−^ mice, which have low platelet counts but normal GPIbα expression in platelets (Figure S2B), and found that 10aa^−/−^ platelet transfusion significantly extended survival compared with WT (Figure 2F). These data demonstrate that the absence of the GPIbα cytoplasmic tail reduces bacteria-induced platelet activation.

### 2.3. The GPIbα Cytoplasmic Tail Exacerbates Platelet Aggregation in S. aureus-Infected Mice

**Figure 3:**
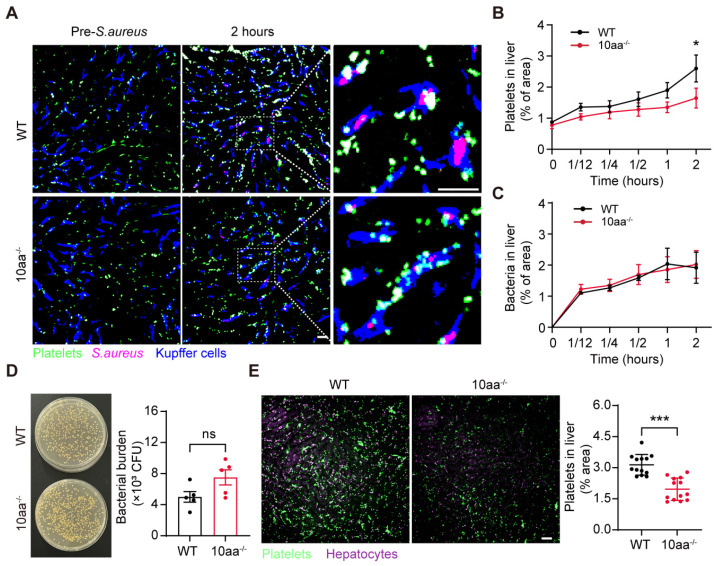
The GPIbα cytoplasmic tail exacerbates platelet aggregation in *S. aureus*-infected mice. (**A**) Representative intravital microscopy (IVM) images of WT and 10aa^−/−^ platelets (DyLight649-GP1bβ, Green), *S. aureus* (PH-red, Red) and Kupffer cells (F4/80, Blue) in the liver at 30 min post-injection (*n* = 3 independent experiments). Scale bars: 25 μm. (**B**,**C**) Quantitative analysis of the area of platelets and bacteria in the liver at different time points (*n* = 3 of mice). (**D**) Bacterial burden in blood at indicated time points (4 h) (*n* = 5 mice per group). (**E**) Representative intravital microscopy (IVM) images and quantitative analysis of platelet aggregates (DyLight649-GP1bβ, Green) in the WT and 10aa^−/−^ liver after 24 h injection of *S. aureus* (*n* = 3 mice per group of different visions). Scale bars: 25 μm. Differences between two groups were assessed using two-way ANOVA test (**B**,**C**) and unpaired two-tailed Student’s *t*-test (**D**,**E**). Data are shown as the mean ± SEM from three or five independent experiments. * *p* < 0.05; *** *p* < 0.001; ns, no significance.

Based on the above findings, we hypothesized that *S. aureus* may activate platelets to incite immunothrombosis during bacterial sepsis in vivo. To inform an understanding of platelet clearance and microvascular thrombosis formation induced by *S. aureus* in situ, we utilized spinning intravital imaging microscopy to visualize bacteria-platelet-Kupffer cell interactions within the hepatic vasculature following infection of 10aa^−/−^ and WT mice with 1 × 10^8^ CFU of *S. aureus*. We found that the platelet count was lower in 10aa^−/−^ mice after inoculation with *S. aureus* at different time points (Figure 3A,B). However, the bacterial load in the liver remained similar between the two groups (Figure 3C). As observed, platelets formed stable aggregates around captured *S. aureus* on the surface of Kupffer cells to facilitate bacterial elimination (Figure 3A). To further explore whether *S. aureus*-induced decreased 10aa^−/−^ platelet activation affects bacteria clearance, we found that the bacterial burden in the blood was slightly higher in 10aa^−/−^ mice than in WT mice at different times (Figure 3D and Figure S2C). The activation of platelets caused by bacterial infection is a continuous process. We found that WT mice developed more substantial platelet aggregates than 10aa^−/−^ mice at 24 h post-infection (Figure 3E). These data demonstrate that the platelet GPIbα cytoplasmic tail deficiency could protect infected mice from excessive platelet clearance and septic thrombosis.

### 2.4. MPαC Recovers Platelet Aggregation of 10aa^−/−^ Mice in Bacterial Sepsis

**Figure 4:**
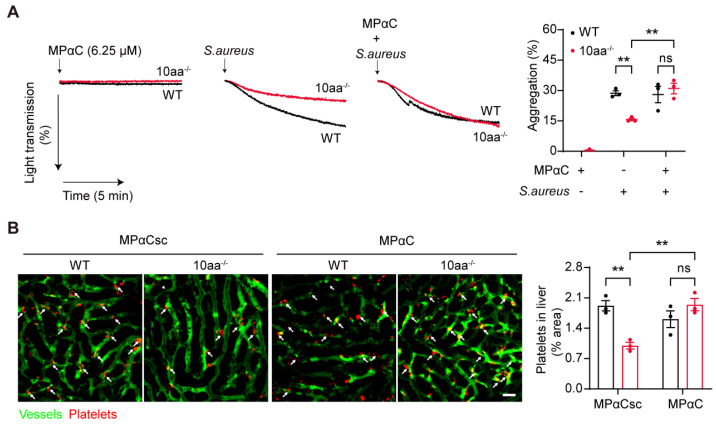
MPαC recovers platelet aggregation of 10aa^−/−^ mice in bacterial sepsis. (**A**) Aggregation and quantification plots of WT and 10aa^−/−^ murine platelets treated with *S. aureus* with or without MPαC, (6.25 μM) (*n* = 3 independent experiments). (**B**) Representative images and quantification of IVM in the WT and 10aa^−/−^ livers intravenous injection of MPαCsc/MPαC before *S. aureus* infection after 24 h (*n* = 3 mice), Platelets: (DyLight649-GP1bβ, Red), Vessels: (FITC-dextran 2000, green), Scale bars: 25 μm. Differences between two groups were assessed using two-way ANOVA test (**A**,**B**). Data are shown as the mean ± SEM from three or five independent experiments. ** *p* < 0.01; ns, no significance.

To further explore the effect of the GPIbα cytoplasmic domain in sepsis, we applied MPαC, a peptide corresponding to the C-terminal 14-3-3ζ binding sequence. As a control, we used (MPαCsc), a peptide whose sequence is randomly arranged. Our previous study found that MPαC (6.25 μM) rescued agonists-induced 10aa^−/−^ platelet activation [28]. Notably, the MPαC concentration (6.25 μM) did not induce washed murine platelet aggregation but instead restored *S. aureus*-induced 10aa^−/−^ platelet aggregation to the level of WT platelets (Figure 4A). These membrane-permeable peptides were incubated with platelets to mimic the elevated cytoplasmic domain of GPIbα in platelets. Compared with WT mice, the platelet aggregates of 10aa^−/−^ mice infected with *S. aureus* were rescued after venous injection with MPαC (6.25 μM) (Figure 4B). These findings further validated that the platelet GPIbα cytoplasmic tail significantly impacts sepsis-induced thrombosis.

### 2.5. The GPIbα Cytoplasmic Tail Regulates S. aureus-Induced Platelet Activation by the PKC-Dependent Pathway

**Figure 5:**
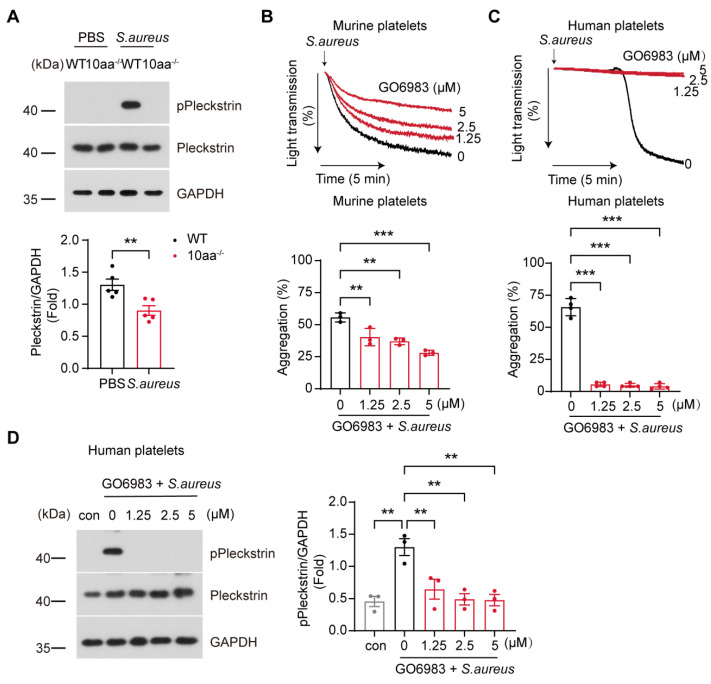
The GPIbα cytoplasmic tail regulates *S. aureus*-induced platelet activation by the PKC-dependent pathway. (**A**) Representative blots and quantification of the phosphorylation levels of PKC substrate (pleckstrin) in WT and 10aa^−/−^ platelets treated with *S. aureus* (*n* = 5 independent experiments). (**B**,**C**) The effect of PKC inhibitor GO6983 on washed murine (**B**) and human (**C**) platelets aggregation induced by *S. aureus* (*n* = 3 independent experiments). (**D**) The level of phosphorylation of PKC substrate (pleckstrin) in human platelets pre-treated with GO6983 stimulated by *S. aureus* (*n* = 3 independent experiments). Two-way ANOVA test in (**A**). One-way ANOVA followed by Dunnett’s post hoc test in (**B**–**D**). Data are shown as the mean ± SEM. ** *p* < 0.01; *** *p* < 0.001; ns, no significance.

Our previous research showed that PKC activity decreases in 10aa^−/−^ platelets and that the GPIbα cytoplasmic tail regulates PKC activity by sequestering 14-3-3ζ [28]. Consistently, in this study, we selected GAPDH as the loading control. We found that 10aa^−/−^ platelets decreased the phosphorylation of pleckstrin induced by *S. aureus*, and the pleckstrin did not change (Figure 5A). In addition, GO6983, a PKC inhibitor, suppressed *S. aureus*-induced washed murine platelet aggregation in a dose-dependent manner (Figure 5B), but a lower concentration (1.25 μM) of GO6983 completely inhibited human platelet aggregation (Figure 5C) and pleckstrin phosphorylation (Figure 5D), suggesting that human platelets may be more sensitive to PKC inhibitors. These data indicate that the GPIbα cytoplasmic tail may promote PKC activity to regulate bacteria-induced platelet activation.

### 2.6. Inhibition of PKC Protects Mice from S. aureus-Induced Platelet Clearance, Thrombosis, and Prolongs Survival

**Figure 6:**
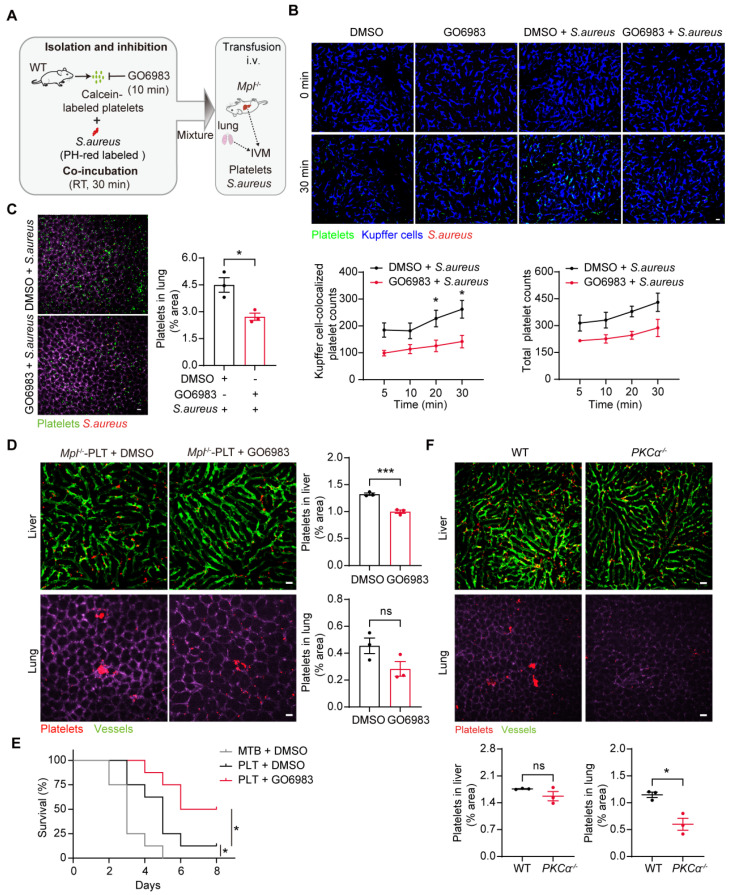
Inhibition of PKC protects mice from *S. aureus*-induced platelet clearance and thrombosis and prolongs survival. (**A**,**B**) The effect of PKC inhibitors on the accumulation of calcein-labeled and *S. aureus*-bound platelets in murine liver observed by intravital confocal microscopy (*n* = 3 independent experiments), platelets: (DyLight649-GP1bβ, Green), *S. aureus*: (PH-red, Red), Kupffer cells: (F4/80, Blue), Scale bars: 25 μm. (**C**) Representative images and quantification of IVM in the *Mpl*^−/−^ lung transferred with a mixture of the GO6983-treated platelet and *S. aureus* (*n* = 3 independent experiments), platelets: (DyLight649-GP1bβ, Green), *S. aureus*: (PH-red, Red), Scale bars: 25 μm. (**D**) The effect of GO6983 on *S. aureus*-induced platelet aggregates formation after 24 h observed by IVM, Scale bars: 25 μm. (**E**) The platelets pre-treated with or without GO6983 were transfused into *Mpl*^−/−^ mice after being injected with *S. aureus* (1.5 × 10^8^ CFU). Survival rate was observed for up to 8 days (*n* = 8 mice per genotype). (**F**) Representative images and quantification of IVM in the WT and *pkcα*^−/−^ livers and lung injected with *S. aureus* after 24 h (*n* = 3 mice per group of different visions), platelets: (DyLight649-GP1bβ, Red), Vessels: (FITC-dextran 2000, green), Scale bars: 25 μm. Two-way ANOVA test in (**B**). Unpaired two-tailed Student’s *t*-test in (**C**,**D**,**F**). Log-rank (Mantel–Cox) test in (**E**). Data are shown as the mean ± SEM. * *p* < 0.05, *** *p* < 0.001; ns, no significance.

To investigate the role of platelet-specific PKC in sepsis and to minimize the influence of endogenous murine platelets, we transfused a mixture of the bacteria and platelets, with or without a PKC inhibitor, into *Mpl*^−/−^ mice (Figure 6A). Using spinning disk confocal microscopy, we observed that fewer GO6983-treated platelets were phagocytosed by Kupffer cells in the liver (Figure 6B, Videos S1 and S2). We also observed platelet aggregates and bacteria-platelet interaction in the lungs. GO6983 inhibited platelet aggregate formation (Figure 6C). In addition, the GO6983-treated platelets reduced septic thrombosis in both the livers and the lungs of *Mpl*^−/−^ mice at 24 h post-infection (Figure 6D, Videos S3 and S4). Given the potential therapeutic value of PKC inhibitors, we transfused GO6983-treated platelets into *Mpl*^−/−^ mice 24 h after infection with a lethal dose (1.5 × 10^8^ CFU) of *S. aureus*. We found that GO6983-treated platelets significantly extended the lifespan of the mice (Figure 6E).

Multiple PKC subtypes participate in regulating macrophage activation, platelet-leukocyte interaction, and apoptosis of immune cells during sepsis [29,30,31]. PKCα plays a major role in thrombus formation [32]. To further verify the crucial role of PKCα in bacteria-induced platelet activation in vivo, a PKCα knockout mouse (*pkcα*^−/−^) infection model was established via intravenous injection of *S. aureus*. Compared with the wild type, the bacteria-induced platelet aggregation was slightly decreased in the livers of *pkcα*^−/−^ mice; the septic thrombus was significantly decreased in the lungs (Figure 6F), demonstrating that platelets PKCα inhibition prevented lung thrombosis formation to a certain extent. These data demonstrate that platelet PKC inhibition reduces bacteria-induced platelet clearance and aggregate formation both in the liver and lung in vivo.

## 3. Discussion

In our study, we demonstrated that the GPIbα cytoplasmic tail aggravated organ injury, platelet clearance, and septic thrombosis, ultimately leading to mortality. We also uncovered a new mechanism by which GPIbα interacts with 14-3-3ζ to regulate bacteria-induced platelet activation, which further affects PKC activation. These findings imply that blocking the platelet GPIbα cytoplasmic tail or inhibiting platelet PKC activity has beneficial effects, suggesting a potential therapeutic strategy in septic thrombosis.

Mice deficient in full-length GPIbα displayed Bernard–Soulier syndrome, characterized by a reduced number of circulating platelets [33], which is insufficient for exerting antibacterial effects. While 10aa^−/−^ mice exhibited slightly decreased platelet counts, their survival was significantly extended in Gram-positive bacterial infection. This phenomenon was weakened in the Gram-negative bacterial infection model. More importantly, the levels of pro-inflammatory factors and liver damage were markedly reduced in 10aa^−/−^ mice (Figure 1). These data indicate that the absence of the GPIbα cytoplasmic tail improves inflammation conditions.

Platelets are known to play a dual role in immune responses. Sepsis-induced thrombosis is a challenge in clinical treatment [34]. Growing evidence indicates that the platelet receptor GPIbα plays a crucial role in regulating various inflammatory thrombotic diseases [35,36]. Platelet production or function destruction could affect platelet-bacteria or platelet-immune cell interaction to trap and clear bacteria, potentially facilitating bacterial dissemination [37,38]. Moreover, *S. aureus* can directly interact with GPIbα or bind to VWF, which then interacts with GPIbα to trigger platelet aggregation [39,40]. The proportion of platelet-bacteria interaction in 10aa^−/−^ mice was similar to that in WT mice; however, bacteria-induced platelet aggregation was decreased in vitro. Furthermore, the absence of the GPIbα domain reduced *S. aureus*-induced intravascular thrombus formation in the liver, but the addition of MPαC (6.25 μM), which compensates for the lack of the GPIbα cytoplasmic domain in 10aa^−/−^ platelets, exacerbated 10aa^−/−^ mouse platelet aggregation in vitro and septic thrombosis in vivo. However, Yin H et al. found that MPαC significantly inhibits LPS-induced microvascular thrombosis [20]. Of note, MPαC (6.25 μM) has little inhibitory effect on septic thrombosis in WT mice during *S. aureus* infection, indicating that the impact of different MPαC concentrations on *S. aureus*-induced bacteremia differs from that of LPS-induced endotoxemia. In sepsis, thrombocytopenia is a frequent complication, and it is closely associated with increased mortality [27,41]. The deficiency of the GPIbα cytoplasmic domain inhibits platelet clearance and alleviates thrombocytopenia during bacterial infection. Therefore, our data demonstrate that the GPIbα cytoplasmic tail is essential for bacterial-induced platelet activation, clearance, and thrombosis.

Current clinical antiplatelet drugs include P2Y12 inhibitors, glycoprotein GPIIb/IIIa receptor antagonists, and serotonin (5-HT) receptor antagonists [42]. These antiplatelet drugs not only inhibit platelet activation and aggregation, thereby improving the hypercoagulable circulation state during sepsis, but also exert anti-inflammatory effects [43]. Due to its key role in initiating thrombosis, GPIbα emerges as an ideal target for anti-thrombotic drug development, which has been conducted for almost forty years [44,45]. However, up to now, no anti-GPIbα drug has been developed successfully. Therefore, exploring the role of the platelet GPIbα cytoplasmic domain in the pathogenesis of sepsis to identify new antiplatelet drugs may facilitate the effective treatment of sepsis-induced thrombosis and thrombocytopenia. The PKC-dependent pathway is involved in the activation of bacterial or viral infection-induced innate immunity [31,46]. We found that the absence of the GPIbα cytoplasmic tail reduced pleckstrin phosphorylation in response to bacterial stimulation, and that inhibiting PKC-dependent platelet activation effectively prevented bacteria-induced platelet aggregation, clearance, and septic thrombosis both in vitro and in vivo. PKCα ablation is involved in hepatic metabolism and failure during sepsis [47], which may further affect septic thrombosis. Inhibition of PKCα attenuates LPS-induced acute lung injury [48]. In our study, decreased thrombosis was observed in the lungs rather than in the liver of PKCα-deficient mice, an effect unlike the effects of the platelet PKC inhibitor. Nevertheless, our results demonstrate the potential benefits of PKC inhibitory platelet transfusion in the treatment of the associated thrombotic inflammation and improvement of the prognosis of sepsis. The effects of platelet PKCα on bacterial load and spread need to be further investigated.

In conclusion, our study reveals the crucial role of the GPIbα cytoplasmic tail and PKC in regulating bacteria-induced platelet activation and clearance. Furthermore, targeting the interaction between GPIbα and 14-3-3ζ or the inhibition of the platelet PKC present potential therapeutic targets for treating septic thrombosis.

## 4. Materials and Methods

### 4.1. Reagents

Myristoylated MPαCsc (C13H27CONH-LSIpSYGSHR) and peptides MPαC (C13H27CONH-SIRYSGHpSL) were synthesized by Sangon Biotech (Shanghai, China). *S. aureus* (ATCC29213) and *E. coli* (ATCC25922) were cultured in our laboratory. Mouse TNFα and IL-6 ELISA Kit were purchased by MULTI SCIENCES (Hangzhou, China). ALT (C009-2-1) and AST (C010-2-1) Assay Kit came from Jiancheng (Nanjing, China). FITC-conjugated rat anti-mouse P-selectin (clone Wug. E9, M130-1), Dylight 649-conjugated anti-GPIbβ (X649) antibodies and PE-conjugated rat anti-mouse Integrin (clone JON/A) came from Emfret Analytics (Eibelstadt, Germany). GO6983 (HY-13689) and Calcein-AM (HY-D0041) were from MedChemExpress (Monmouth Junction, NJ, USA). Rabbit antibodies against Phospho-(Ser) PKC Substrate (2261), GAPDH (5174), and 10 × cell lysis buffer (9803) came from Cell Signaling Technology (Beverly, MA, USA). PLEK Polyclonal antibody (12506-1-AP) was from Proteintech (Wuhan, China). PE-conjugated anti-F4/80 (123109) antibodies were from Biolegend (San Diego, CA, USA). 4 × SDS-PAGE loading buffer (with DTT) was from Beyotime. LB Broth was from Solarbio (Beijing, China). Plate Count Agar was from Hope Bio-technology (Qingdao, China). Thrombin and fibrinogen were purchased from Sigma-Aldrich (St. Louis, MO, USA).

### 4.2. Human Studies

All experiments using healthy volunteer subjects were approved by the Ethics Committee of The First Affiliated Hospital of Soochow University and performed following the Declaration of Helsinki. Written informed consent has been obtained from the volunteers to publish this paper.

### 4.3. Animals

C57BL/6J wild-type mice were bought from JOINN Laboratories (Suzhou, China). The 10aa^−/−^ (lacking 10 amino acids at the C-terminus of GPIbα) mice were constructed by our laboratory. B6/JGpt-*Prkca*^em1Cd5428^/Gpt mice (T027858) were acquired from GemPharmatech Co., Ltd. (Nanjing, China). *Mpl*^−/−^ mice were provided by Junping Wang (Third Military Medical University, Chongqing, China). Furthermore, all experimental animals utilized in this work were raised in the SPF environment at Soochow University. Twelve or eight male mice were used for survival experiments. Other experiments involved more than three mice, and the male and female sexes were balanced (6–8 weeks). All animal experiments were conducted using sodium pentobarbital or Isoflurane gas anesthesia and euthanized. Additionally, The First Affiliated Hospital of Soochow University and Ethics Committee approved all animal research.

### 4.4. Platelets Preparation and Staining

Washed platelets: Whole blood was drawn from the inferior vena cava of wild type (WT) and 10aa^−/−^ mice and anticoagulated with a 1:7 volume of acid–citrate–dextrose (ACD: 2.5% trisodium citrate, 2.0% D-glucose, 1.5% citric acid). Platelet-rich plasma (PRP) was collected by centrifugation at 200× *g* for 10 min. Platelets were washed twice with CGS buffer (0.123 M NaCl, 0.033 M D-glucose, 0.013 M trisodium citrate, pH 6.5), ultimately resuspended in modified Tyrode’s buffer (MTB) (2.5 mM Hepes, 150 mM NaCl, 2.5 mM KCl, 12 mM NaHCO_3_, 5.5 mM D-glucose, 1 mM CaCl_2_, 1 mM MgCl_2_, pH 7.4) to concentration of 3 × 10^8^/mL or 10 × 10^8^/mL, and allowed to incubate at 22 °C for 2 h.

**Platelets staining**: Murine platelets were washed twice with CGS, labeled with calcein-AM (5 μg/mL) at RT for 15 min, and resuspended in MTB to a final concentration of 1 × 10^9^/mL.

### 4.5. S. aureus and E. coli Culture Conditions and Staining

Bacteria were grown in Luria-Bertani (LB) media at 37 °C for 12 h while shaking (200 rpm). Bacterial culture supernatants were centrifugated at 2500 rpm for 10 min and for CFU counting on the LB agar plates. We used a Eppendorf BioPhotometer (Eppendorf AG, Hamburg, Germany) to detect starting OD (OD (*E. coli*) = 0.683; OD (*S. aureus*) = 0.492), and stored them in 30% glycerin/LB mixture at −80 °C. Alternatively, *S. aureus* or *E. coli*, labeled with PH-red (1:2000, dark at room 30 min), was washed by centrifugation at 2000× *g* for 5 min and resuspended in 1 × PBS (1.5 × 10^8^ CFU/mouse).

### 4.6. Aggregation

For the platelet aggregation assay, human platelets or washed wild type (WT) and 10aa^−/−^ platelets (3 × 10^8^/mL), pre-treated with GO6983 (1.25 µM, 2.5 µM, 5 µM) or MPαC (6.25 µM), were used in response to *S. aureus* at 37 °C under 1200 rpm stirring. Platelet aggregation was monitored using a Chrono-Log aggregometer (CHRONO-LOG700, Pennsylvania, USA) over 10 min.

### 4.7. Platelet Spreading and Clot Retraction

WT and 10aa^−/−^ platelets were prepared as described previously. Platelets (0.2 × 10^8^/mL) were stimulated with PRP4-AP (40 µM) and spread on immobilized fibrinogen at 37 °C for 2 h. Platelets were stained with rhodamine-conjugated phalloidin and visualized with a LEICA TCS SP8 confocal microscope (Wetzlar, Germany) with a 63 × oil immersion lens. Five images were taken at random and evaluated with ImageJ Version 1.51j8.

Clot retraction (4 × 10^8^/mL) was conducted in the presence of thrombin (0.05 U/mL) and fibrinogen (150 µg/mL). Images were taken at different time points, and the area of clot retraction was measured using ImageJ.

### 4.8. Flow Cytometry

Blood samples were collected from the post-glomus venous plexus of wild-type and 10aa^−/−^ mice injected with 1.5 × 10^8^ CFU *S. aureus* after 4 h, and then stimulated with PAR4-AP at 37 °C for 30 min. The platelets (3 × 10^8^/mL) were labeled by FITC-conjugated P-selectin antibody (mouse) and PE-conjugated JON/A antibody (mouse) for 15 min. The levels of P-selectin exposure and JON/A binding were measured by a flow cytometer (FC 500; Beckman-Coulter, Brea, CA, USA).

### 4.9. ELISA

The whole blood, anti-coagulated with EDTA, was collected from the post-glomus venous plexus of WT and 10aa^−/−^ mice (6~8-week-old) i.v. injected with *S. aureus* at different points of time (0 h, 1 h, 2 h, and 4 h for IL-6 and TNFα measurements; 0 h and 24 h for aspartate aminotransferase and alanine aminotransferase measurements) and centrifugated at 1000× *g* for 30 min. The levels of IL-6 and TNFα were performed according to the manufacturer’s protocol. Briefly, 20 μL of plasma per sample was mixed with 80 μL of reaction buffer, and incubated at 100–300 rpm for 1.5 h at RT. We then washed the plate and added HRP to incubate for 30 min. The activity was determined by an ELISA reader at an absorbance of 450 nm.

The aspartate aminotransferase and alanine aminotransferase levels were measured according to the manufacturer’s protocol. Briefly, 5 μL of plasma per sample was mixed with 20 μL of matrix fluid and dinitrophenylhydrazine at 37 °C for 30 min. Finally, 200 μL 0.4 M NaOH was added to stop the reaction and detected by an ELISA reader at an absorbance of 510 nm.

### 4.10. Survival Studies

WT, 10aa^−/−^ or *Mpl*^−/−^ mice were given Isoflurane gas anesthesia and intravenously injected with 1.5 × 10^8^ colony-forming units (CFU) *S. aureus* or *E. coli* and monitored for 8 days for survival studies. The septic animal model establishment complied with the Minimum Quality Threshold in Preclinical Sepsis Studies (MQTiPSS) criteria [49]. After observation, we euthanized the mice.

### 4.11. Bacterial Load in Blood

The blood was collected from the post-glomus venous plexus of WT and 10aa^−/−^ mice injected with *S. aureus* at 4 h. Whole blood was diluted with sterile water and plated on agar, then incubated overnight at 37 °C. The next day, colony formation was counted.

### 4.12. Western Blot

Washed WT and 10aa^−/−^ platelets or human platelets (3 × 10^8^/mL), pre-treated with or without GO6983, were stimulated with *S. aureus* for 1 h at 37 °C and lysed with an equal volume of lysis buffer on ice for 30 min. All samples were evaluated by western blotting.

### 4.13. Platelet Count

WT and 10aa^−/−^ mice were preinjected intravenously (i.v.) with *S. aureus* (1.5 × 10^8^ CFU). The whole blood, anticoagulated with 3.8% trisodium citrate, was collected from the post-glomus venous plexus at indicated time points. Platelets were counted by a Sysmex XP-100 Hematologic Analyzer (XP-100, Kobe, Japan).

### 4.14. Platelets Transfusion Assay

Washed WT platelets were incubated with or without PKC inhibitors (5 μM GO6983) and then transfused to *Mpl*^−/−^ recipient mice through the post-glomus venous plexus (1 × 10^9^ platelets in 100 μL MTB).

### 4.15. Platelet and S. aureus Adhesion

For detecting platelet and bacterial interactions in vitro, WT and 10aa^−/−^ RPR (3 × 10^8^/mL) were collected from the post-glomus venous plexus and co-cultured with PH-red-labeled *S. aureus* at 37 °C for 30 min. Then the mixture was centrifugated at 1200 rpm for 5 min.

For detecting platelets and bacteria interaction in vivo, the PH-red-labeled *S. aureus* (1.5 × 10^8^ CFU) were injected into WT and 10aa^−/−^ mice and blood was collected through the post-glomus venous plexus at 30 s. The blood was then fixed in 4% PFA and subsequently blocked in 5% BSA/PBS at RT for 1 h. Anti-mouse CD42b (Emfret, clone Xia G5, 10 μg/mL) was incubated overnight. Confocal Microscopy1003 lens 63 × oil lens was used to randomly obtain images. The images were analyzed using Imaging J Version 1.51j8.

### 4.16. Intravital Microscopy

WT, 10aa^−/−^, or *Pkcα*^−/−^ mice were infected with *S. aureus* (1 × 10^8^ CFU/mouse) and i.v. injected with Dylight 649-conjugated anti-GPIbβ (X649) antibody to label platelets. The livers and lungs of these mice were observed by spinning disk confocal microscopy (VIVO Intravital Imaging System, Intelligent Imaging Innovations, Denver, USA) using an sCMOS camera (ORCA Flash4.0, Hamamatsu, Japan).

Calcein-labeled washed WT platelets (1 × 10^9^/mL) were pre-treated with or without PKC inhibitors GO6983 (5 μM) for 10 min at RT and then stimulated by *S. aureus* (platelet: bacteria counts = 1:4) at RT for 30 min. The *Mpl*^−/−^ recipient mice were anesthetized by sodium pentobarbital, then i.v. injected with PE-conjugated anti-F4/80 antibody. Ten min later, the liver of the mouse was exposed by surgical operation, and in vivo imaging was observed. The bacteria-platelet mixture was injected into the tail catheter to record the sequestration of platelets in the liver. These processes were monitored in real time by spinning disk confocal microscopy. After observation, the mice were euthanized.

### 4.17. Statistics Analysis

GraphPad Prism 9.0.0 software was used for statistical analysis. Numeric data were analyzed using One-way (for a single variant) or Two-way (for multiple variants) ANOVA, respectively. Two groups were compared by two-tailed Student’s *t*-tests. Survival curves were compared using a log-rank test with a hazard ratio. All data are presented as the mean ± SEM. Different *p*-values are indicated as * *p* < 0.05, ** *p* < 0.01, and *** *p* < 0.001 and considered statistically significant.

## 5. Conclusions

In summary, these data reveal that the GPIbα cytoplasmic domain promotes bacteria-induced platelet activation, clearance, and septic thrombosis by regulating PKC activation. Therefore, inhibiting platelet activation with the PKC inhibitor appears to be a novel anti-bacterial strategy.

## Data Availability

The original contributions presented in this study are included in the article/Supplementary Materials. Further inquiries can be directed to the corresponding author(s).

## Supplementary Materials

The supporting information can be downloaded at: https://www.mdpi.com/article/10.3390/ijms252111548/s1.
