# Supplementary material for: Platelet Glycoprotein Ibα Cytoplasmic Tail Exacerbates Thrombosis During Bacterial Sepsis
Source: Int J Mol Sci. 2024 Oct 27;25(21):11548. doi: 10.3390/ijms252111548 (PMC11546206; doi:10.3390/ijms252111548)
Supplement: Supplementary file 1 [file ijms-25-11548-s001.zip › ijms-3264060-supplementary.pdf]

SUPPLEMENTAL FIGURES

Platelet Glycoprotein Ibα Cytoplasmic Tail Exacerbates Thrombosis During Bacterial Sepsis

Yue Xia †, Chenglin Sun †, Kangxi Zhou, Jie Shen, Jiaojiao Li, Qiuxia Huang, Jiahao Du, Sai Zhang, Kang Sun, Renping Hu, Rong Yan * and Kesheng Dai *

Jiangsu Institute of Hematology, Cyrus Tang Medical Institute, Suzhou Medical College of Soochow University, NHC Key Laboratory of Thrombosis and Hemostasis, National Clinical Research Center for Hematological Diseases, Suzhou 215123, China

* Correspondence: yanrong2622@suda.edu.cn (R.Y.); kdai@suda.edu.cn (K.D.); Tel./Fax: +86-512-67781370 (K.D.)

† These authors contributed equally to this work.

Supplemental Figures and Figure Legends

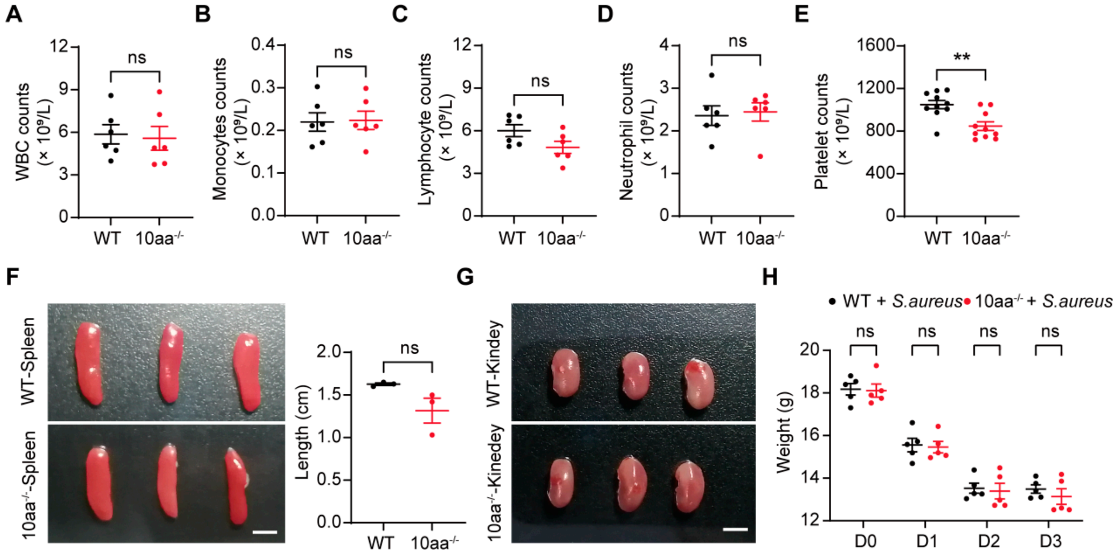

Figure S1. Peripheral blood cell counts and organ damage of 10aa$^{-/-}$ mice. (**A**-**E**) WBC, Monocytes, Lymphocytes, Neutrophils and Platelet counts in WT and 10aa$^{-/-}$ mice (n = 6 mice). (**F** and **G**) WT and 10aa$^{-/-}$ mice were killed, and the spleen (**F**) and kidney (**G**) were taken photos after *S.aureus* injection at 24 hours (n = 3 mice), Scale bar: 0.5 cm. (**H**) The weight of WT and 10aa$^{-/-}$ mice infected *S.aureus* (n = 5 independent experiments). Two-tailed Student's t test in (**A**-**F**) and Two-way ANOVA followed by Bonferroni's post hoc test in (**H**). Data are shown as mean ± SEM. ***P* < 0.01. ns, no significance.

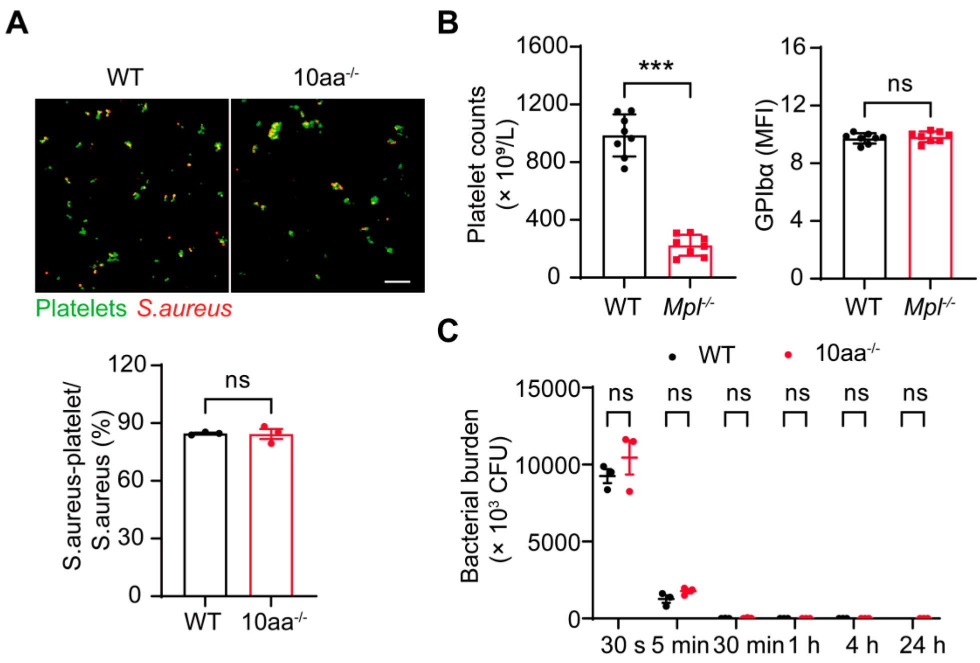

Figure S2. The effects of GPIbα cytoplasmic tail on platelet-bacteria interaction and bacteria clearance. (**A**) Representative confocal images and quantification of the interaction of PH-Red-labeled-*S.aureus* with WT and $10aa^{-/-}$ platelets (n = 3 mice per genotype). Scale bar: 10 µm. (**B**) Platelet counts and GPIbα expression in *Mpl*$^{-/-}$ mice (n = 8 mice per genotype). (C) Bacterial burden in blood at different time points (30 s、5 min、30 min、1 h、4 h、24 h) (n = 3 mice per group). Two-tailed Student's t test in (**A** and **B**). Differences between two groups were assessed using two-way ANOVA test (**C**). Data are shown as mean ± SEM. ***P < 0.001. ns, no significance.

Video legends

Video S1. Effect of DMSO on *S.aureus*-induced platelets clearance in the liver of *Mpl*$^{-/-}$ mice. Liver intravital microscopy in *Mpl*$^{-/-}$ mouse i.v injection of *S.aureus*-incubated with washed WT platelets, pretreated with DMSO (150 µL

mixture/mouse i.v.). Platelets labeled with calcein-AM (Green); *S.aureus* labeled with PH-Red (Red); Kupffer cell labeled with F4/80 (Blue).

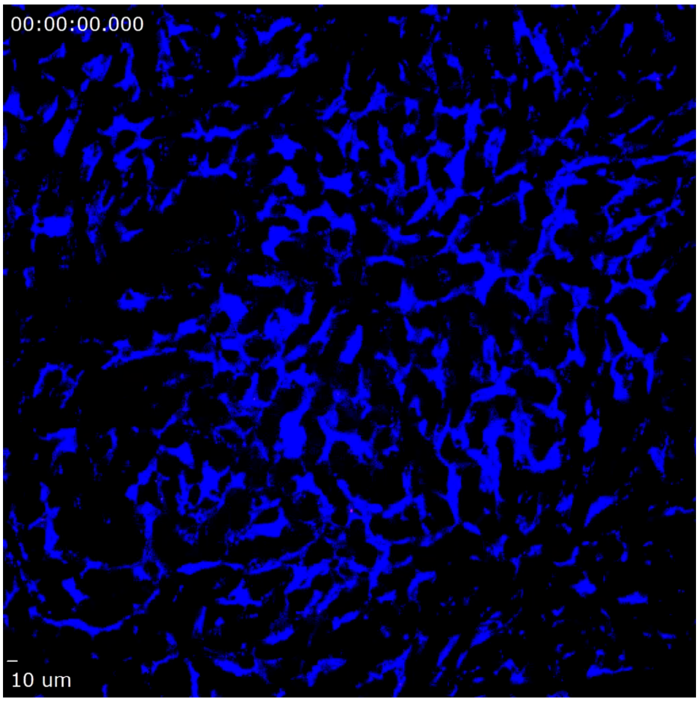

Video S2. Effect of GO6983 on *S.aureus*-induced platelets clearance in the liver of *Mpl*$^{-/-}$ mice. Liver intravital microscopy in *Mpl*$^{-/-}$ mice i.v injection of *S.aureus*-incubated with washed WT platelets, pretreated with GO6983 (150 μL mixture/mouse i.v.). Platelets labeled with calcein-AM (Green); *S.aureus* labeled with PH-Red (Red); Kupffer cell labeled with F4/80 (Blue).

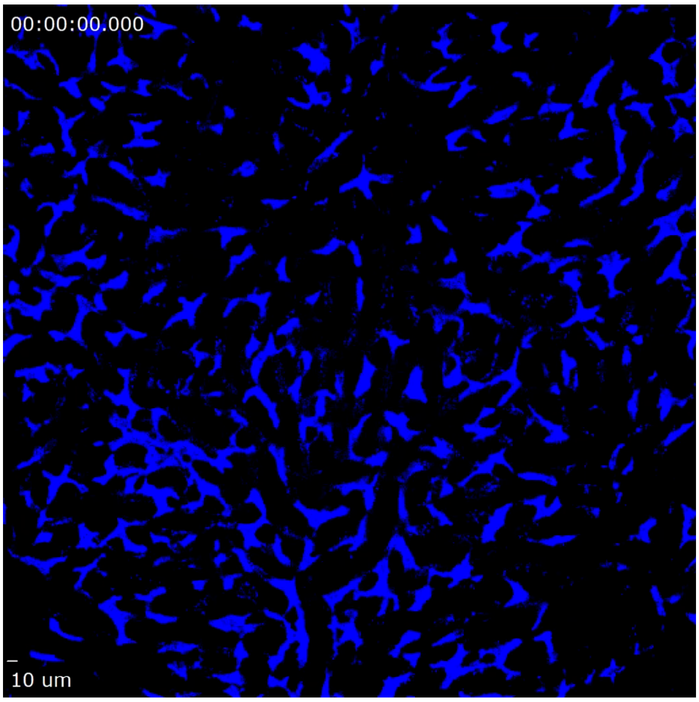

Video S3. Effect of DMSO-treated platelets on *S.aureus*-induced platelets aggregates in the microvasculature of the liver in *Mpl*$^{-/-}$ mice. Visualization of *Mpl*$^{-/-}$ mice infected with *S.aureus* transfusing of WT washed platelets at 24 hours. Platelets labeled with DyLight649-conjugated- anti-GP1bβ (Red); Blood flow labeled with FITC-dextran 2000 (Green).

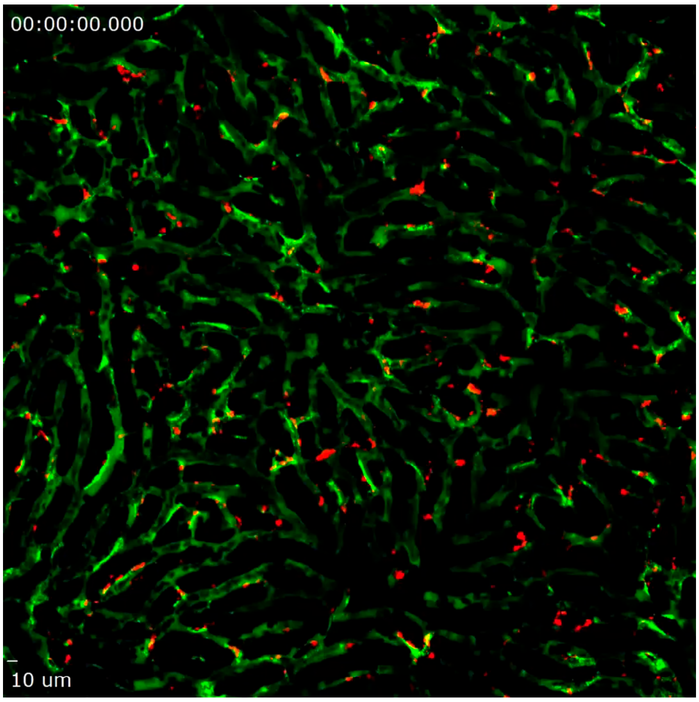

Video S4. Effect of GO6983-treated platelets on *S.aureus*-induced platelet aggregates in the microvasculature of the liver in *Mpl*$^{-/-}$ mice. Visualization of *Mpl*$^{-/-}$ mice infected with *S.aureus* transfusing of WT washed platelets, pretreated with GO6983. Platelets labeled with DyLight649-conjugated-anti-GP1bβ (Red); Blood flow labeled with FITC-dextran 2000 (Green).

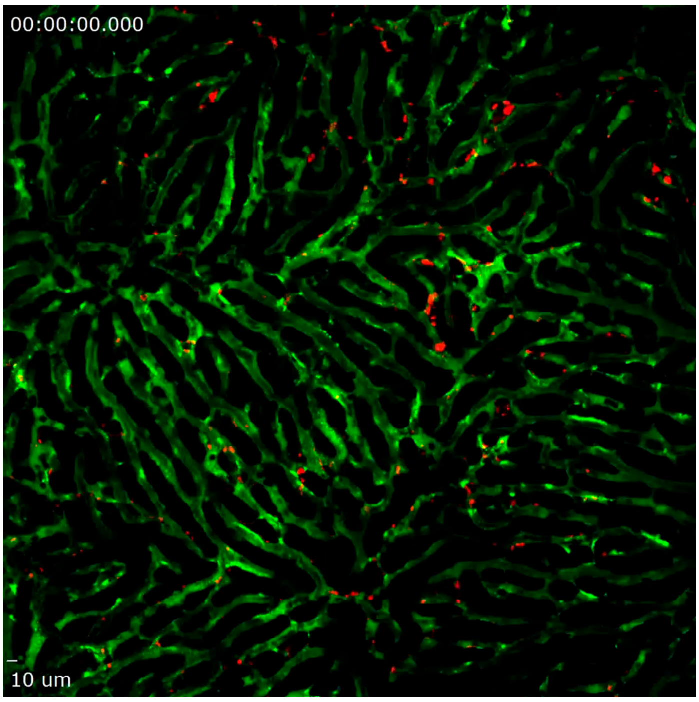
00:00:00.000
10 um
